# Retaining biodiversity in intensive farmland: epiphyte removal in oil palm plantations does not affect yield

**DOI:** 10.1002/ece3.1462

**Published:** 2015-04-29

**Authors:** Graham W Prescott, David P Edwards, William A Foster

**Affiliations:** 1 Department of Zoology, University of CambridgeDowning Street, Cambridge, Cambridgeshire, UK; 2 Department of Animal and Plant Sciences, University of SheffieldSheffield, UK; 3 Centre for Tropical Environmental and Sustainability Science (TESS) and School of Marine and Tropical Biology, James Cook UniversityCairns, Queensland, Australia

**Keywords:** Agriculture, ants, birds, epiphytes, oil palm, South-East Asia

## Abstract

The expansion of agriculture into tropical forest frontiers is one of the primary drivers of the global extinction crisis, resulting in calls to intensify tropical agriculture to reduce demand for more forest land and thus spare land for nature. Intensification is likely to reduce habitat complexity, with profound consequences for biodiversity within agricultural landscapes. Understanding which features of habitat complexity are essential for maintaining biodiversity and associated ecosystem services within agricultural landscapes without compromising productivity is therefore key to limiting the environmental damage associated with producing food intensively. Here, we focus on oil palm, a rapidly expanding crop in the tropics and subject to frequent calls for increased intensification. One promoted strategy is to remove epiphytes that cover the trunks of oil palms, and we ask whether this treatment affects either biodiversity or yield. We experimentally tested this by removing epiphytes from four-hectare plots and seeing if the biodiversity and production of fruit bunches 2 months and 16 months later differed from equivalent control plots where epiphytes were left uncut. We found a species-rich and taxonomically diverse epiphyte community of 58 species from 31 families. Epiphyte removal did not affect the production of fresh fruit bunches, or the species richness and community composition of birds and ants, although the impact on other components of biodiversity remains unknown. We conclude that as they do not adversely affect palm oil production, the diverse epiphyte flora should be left uncut. Our results underscore the importance of experimentally determining the effects of habitat complexity on yield before introducing intensive methods with no discernible benefits.

## Introduction

With the global population expected to increase by 40%, daily per capita calorie intake increasing by 11%, and a shift to a more meat-heavy diet, it is estimated that food production levels in 2050 will need to be 100% higher than those in 2005–2007 (Tilman et al. 2011). Additionally, the International Energy Agency estimates that production of biofuels will treble from 1.3 million barrels of oil equivalent per day (mboe/d) in 2011 to 4.1 mboe/d in 2035 (International Energy Agency 2013). Future pressure to convert natural habitats into cropland to meet these demands is likely to be concentrated in the tropics, where the largest areas of available land, highest projected levels of increase in population and associated food and energy demands, and most favorable climates for many crops and biofuels are located (Laurance et al. 2014).

The expansion of agriculture into tropical frontier forests is one of the major drivers of the global extinction crisis (Gibson et al. 2011). Land-sparing farming is one of the mechanisms proposed to limit further expansion of agriculture and biodiversity loss (Green et al. 2005). This approach maximizes yield on existing farmland, so that global food demands can be met using a minimal amount of agricultural land, thus reducing the need to further convert diverse natural habitats (see Phalan et al. (2011)).

The intensification of agriculture required by a land-sparing approach has, however, been linked to biodiversity declines within agricultural habitats (e.g., Donald et al. 2001; Kremen et al. 2002; Steffan-Dewenter et al. 2007). Intensification generally involves the removal of plant species that compete with crops for light, water, and nutrients – which, in addition to directly diminishing plant diversity, can lower animal species richness and abundance by removing food sources and reducing habitat complexity – and the use of pesticides, which further diminish animal populations (Tscharntke et al. 2005). Species loss can negatively impact key ecosystem functions and services, such as nutrient recycling or pest predation (Tscharntke et al. 2012). Besides reducing the ability of many species to persist within agricultural landscapes, intensification also curtails the ability of species to disperse through the agricultural matrix, exacerbating the effects of habitat fragmentation (Kupfer et al. 2006).

Given that intensification is widely promoted to avoid further loss of natural habitats (Green et al. 2005; Phalan et al. 2011), but can have negative effects on biodiversity and its associated ecosystem services within agricultural landscapes, it is vital to determine which features of habitat complexity can be maintained without compromising productivity. This is especially important in light of the widespread persistence of agricultural intensification practices that decrease biodiversity but perversely have no positive effect on yield. For example, shade trees are often removed from coffee and cacao plantations, but moderate shade cover in these landscapes can support both biodiversity and high yields (Staver et al. 2001; Perfecto et al. 2005; Clough et al. 2011).

Oil palm (*Elaeis guineensis*) is one of the most important tropical crops. It is currently planted on over 16 million hectares (Mha) of tropical land, and over 50% of recent oil palm expansion in Indonesia, Malaysia, and Papua New Guinea occurred at the expense of forest (Gunarso et al. 2013). Most taxa that have been surveyed are less diverse and abundant in oil palm than in forest (Foster et al. 2011), and the expansion of oil palm is thus a major contributor to the tropical extinction crisis. The expansion of oil palm is also set to continue: Corley (2009) estimated that the global demand for palm oil will increase fivefold from 37 megatonnes (Mt) in 2006/7 to 120–156 Mt in 2050. At current yields, this will require an additional 19.1 Mha of palm oil plantations, while even under improved yields, 12 Mha (c.f. 16 Mha currently planted) will still be required to meet medium estimates of future vegetable oil demand (Corley 2009).

Increasing oil palm yield is therefore key to reducing demand for further land. Some of this yield gap will be met via widespread implementation of best management practices with regard to planting, harvesting, and nutrient regimes (Donough et al. 2009). However, it is also likely that management will increasingly focus on removing competing vegetation in the form of herbaceous understory and epiphytes on oil palm trunks. This vegetation can be diverse: for instance, Piggott (1980) recorded 44 species of epiphytic ferns in West Malaysian oil palm plantations. However, Piggott also found that epiphytes were regularly removed from 39% of mature plantations sampled, and epiphyte removal is presently recommended in several management practice guides (Jacquemard 1998; Rankine and Fairhurst 1998; Turner and Gillbanks 2003).

The experimental removal of the understory layer reduced the species richness (but not abundance) of birds in oil palm plantations in Guatemala (Nájera and Simonetti 2010). However, the effects of epiphyte removal have not yet been experimentally tested. Koh (2008) found that epiphyte presence in plantations in Malaysian Borneo was correlated with an increased bird species richness of 1.5 species, whereas Azhar et al. (2011) found that epiphyte presence was not an important predictor of bird species richness in West Malaysia. Subsequent work by Azhar et al. (2013) suggested that lower epiphyte persistence was associated with higher functional diversity of birds (see also Cruz-Angón and Greenberg (2005) for effects of shade tree epiphytes on birds in coffee estates). *Asplenium nidus* ferns are important nesting sites for ants within oil palm plantations, hosting almost as many species of ants (albeit an almost completely different set of species) as their counterparts in forest habitats (Fayle et al. 2010). Yet nothing is known about whether other epiphyte species in oil palm plantations are important for ants.

We asked two fundamental questions about the removal of epiphytes in oil palm plantations. First, does the removal of epiphytes affect biodiversity? We focused on two widespread, functionally important groups: birds and ants. Second, does epiphyte removal alter oil palm yield? These are key questions for identifying which processes will actually increase yield and prevent the unnecessary removal of biodiversity from farmland.

## Materials and Methods

### Sample sites

**Figure 1:**
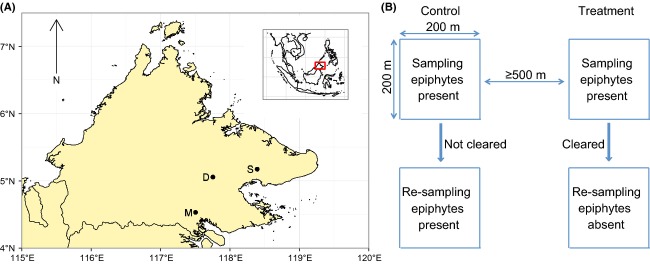
(A) location of our field sites – Danumpalm (D), Mawang (M), and Sabahmas (S) – and (B) overview of our experimental design.

We set up experimental plots in three oil palm estates in Sabah, Malaysian Borneo, a state where 20% of the land in 2010 was used for oil palm cultivation (Gunarso et al. 2013). These estates were Danumpalm (5°03′13.3″N, 117°45′17.3″E; comprising two nearby small holdings, Danumpalm and Kebun Jaya), Mawang (4°31′44.8″N, 117°30′16.1″E), and Sabahmas (5°10′19.7″N, 118°23′36.5″E) (Fig.1A). We carried out our experiments between 2011 and 2013. The palms in our study plots were planted in 1996 (Sabahmas) and 1998–2002 (Danumpalm and Mawang) and are thus representative of mature oil palm (Luskin and Potts 2011). The estates were widely spaced apart and planted on land that once would have been lowland dipterocarp forest. The climate of the area is wet and tropical e.g., mean annual rainfall and temperature of 2822 mm and 26.7°C, respectively (Marsh and Greer 1992).

### Experimental design

In each estate, we set up blocks (eight in total: three in Danumpalm, two in Mawang, and three in Sabahmas) within which our study plots would be located. Each block was separated by at least 5 km, with the exception of two blocks in Danumpalm, which were only separated from each other by 1 km. Each block contained two plots each of 200 m by 200 m in size and separated from each other by at least 500 m. The plots in each block contained palms of the same age. We randomly assigned one plot in each block as the treatment (termed “treatment plots” herein) by coin flip, the other to act as a control (termed “control plots” herein). In control plots, epiphytes were left uncut (the standard management practice in all the estates we sampled), whereas in treatment plots, the epiphytes were cut off by plantation workers using harvesting scythes and machetes. Our experimental design is summarized in Figure1B.

### Time scales of experiment – short term and longer term

We carried out our experiment at different time scales in different sites. In the short-term experiment, we conducted at Danumpalm (three blocks, six plots) and Mawang (two blocks, four plots) we sampled biodiversity data both before clearance and 2 months after the application of a single round of epiphyte clearance in the removal plots.

In the longer-term experiment, we conducted at Sabahmas (three blocks, six plots), the one estate for which we could obtain yield data, epiphyte clearance was repeated every 2 months, and we sampled biodiversity before the first round and 16 months after the first round of clearance started.

### Biodiversity sampling

*Epiphytes* – before clearance, we conducted a full survey from the ground of all the vascular epiphytes (i.e., not including bryophytes) present on each of five palms in each plot. We did not sample non-vascular epiphytes, such as the mosses found on oil palm trunks, because they are not removed as part of management practice. The oil palms we sampled in each plot were at least 30 m apart. Sampled oil palms were 9–17 years old and thus from ground level all vascular epiphytes that protruded from the palm trunk (i.e., adult epiphytes) could be seen easily. Because it can be difficult to discern whether different fronds belong to the same individual fern or not when surveying from the ground, we scored presence–absence for each species at a palm level. We identified ferns using identification guides (Holttum 1954; Piggott 1988) and reference collections at the herbarium at the Forest Research Centre in Sepilok. Botanical experts (Mike Bernadus, Danum Valley Field Centre and Markus Gubilil Forest Research Centre, Sepilok) assisted our identification of angiosperms and ferns, respectively.

*Avifauna* – we conducted three fixed-radius (100 m) 10-min point counts on separate days at the center of each plot between 05:45 and 09:30. The sample size was five control plots and five treatment plots (two of each in Mawang and three of each in Danumpalm) for the short-term experiment, and three control plots and three treatment plots for the longer-term experiment in Sabahmas. We noted all birds seen and heard; we recorded any unfamiliar calls using a Sennheiser SE42 shotgun microphone for subsequent identification by ornithological expert (DPE) and against reference collections (www.xeno-canto.org). Birds estimated to be further than 100 m were not included in the analysis as they would be outside the plots. We excluded domestic chickens (*Gallus gallus*) and birds that flew over plantations without stopping in them (e.g., pacific swallow (*Hirundo tahitica*) and little egret (*Egretta garzetta*)) from the analysis.

*Canopy ants* – we fogged three randomly selected palms within each plot. The palms we sampled were at least 30 m apart, and following Woodcock et al. (2011), we treated them as statistically independent. We fogged fifteen palms in five control plots and fifteen palms in five treatment plots for the short-term experiment, and nine palms in three control plots and nine palms in three treatment plots for the longer-term experiment. We fogged the canopy of each palm for 2 min between 05:45 and 09:00 (as wind is lowest in the early morning) using 0.5% *α*-cypermethrin dissolved in diesel. We collected samples 2 h after fogging to allow enough time for the ants to drop from the canopy. We stored ants in 70% ethanol and identified all worker ants to genus and morpho-species using the Fayle et al. ([Bibr b14]) key (available at http://www.tomfayle.com/Ant%20key.htm).

*Trunk ants* – We collected ants on three oil palm trunks in each plot (the same palms whose canopies we sampled, apart from two plots in one block in Danumpalm for which we were unable to obtain trunk ant samples), by searching for 10 min between 09:00 and 13:00 and collecting any workers with a handheld vacuum cleaner. We sampled the trunks of twelve palms in four control plots and twelve palms in four treatment plots for the short-term experiment, and nine palms in three control plots and nine palms in three treatment plots for the longer-term experiment. Again, ants were stored in 70% ethanol and identified to genus and morpho-species using the Fayle et al. ([Bibr b14]) key (available at http://www.tomfayle.com/Ant%20key.htm).

### Measuring palm oil yield

We were only able to obtain yield data from one estate (Sabahmas). In this estate, we set up one subplot of 140 × 140 m (containing approximately 200 palms – the lot allocated to a single harvester) within each experimental plot (i.e., those in which we sampled biodiversity in the longer-term experiment, *n* - 6). In these subplots, oil palm harvesters recorded the number of fresh fruit bunches harvested and the mass of 20 randomly chosen fresh fruit bunches (the worker measuring the number and mass of fresh fruit bunches was constant for each subplot, but varied among subplots), allowing the mean weight of collected fruit bunches within each plot to be calculated. For each harvesting round within a month at each subplot, we calculated the total mass of fresh fruit bunches produced (tFFB) by multiplying the number of fresh fruit bunches produced in that month (nFFB) by the mean mass of fresh fruit bunches from that round (mFFB). We then summed tFFB for each month at each subplot. We collected these yield data in June 2012, before the first round of epiphyte clearance – which occurred in late June 2012 – and for the subsequent 15 months (July 2012–September 2013), during which epiphytes were removed every 2 months. The last round of removal was at the end of August 2013.

### Statistical analysis

#### Biodiversity

*Epiphytes* – We used the vegan package (Oksanen et al. 2013) in R version 3.03 (R Core Team 2014) to estimated the total number of epiphyte species using sample-based species richness estimation measures (Chao, Jack 1, Jack 2, and Bootstrap) and to plot sample-based species accumulation curves with 95% confidence intervals. We treated each palm as an independent sample (“site”) and performed the same analysis on fern and angiosperm epiphyte communities separately.

*Bird and ant species richness and abundance* – we analyzed bird, canopy ant and trunk ant data in our short-term and longer-term experiments using mixed models in the lme4 R package (Bates et al. 2014) to compare the species richness and abundance between treatment and control plots. Our approach in each case was to compare null models against models also containing treatment as a fixed effect. For example, to test the effects of epiphyte removal on bird species richness in the short-term experiment, the null model was that bird species richness in a plot 2 months after epiphyte clearance was a function of baseline (pre-epiphyte clearance) species richness in that plot, with block nested within estate as random effects. The alternative model against which this would be tested is that the bird species richness of a plot 2 months after clearance was a function of the same factors as the null model (baseline species richness and random effects), as well as treatment (i.e., whether or not epiphytes were cleared in that plot). We used a similar approach for all analyses; we specify below ways in which we modified the approach for different response variables. Within the information theoretical framework of our analysis, the model with the lowest AICc value is deemed to be the best at explaining the data (Burnham and Anderson 2002). We plotted our graphs using the ggplot2 R package (Wickham 2009).

For birds in the short-term experiment, we used the total number of species recorded or the mean abundance at a plot over the 3 days of recording as the response variable. For these response variables, we used linear mixed models (LMMs) with maximum likelihood (ML) estimation and normal error structure, but applied a square-root transformation to meet model assumptions. The null models for species richness and abundance contained baseline (pre-epiphyte removal) species richness or abundance as a fixed effect, and block nested within estate as random effects. We compared this against a model that also contained treatment as a fixed effect.

We took the same approach for birds in the longer-term experiment but because of the small sample size (three control plots and three removal plots), the null models of bird richness and abundance only included block as a random factor to account for any spatial effects. The null models could not include baseline abundance or richness, however, because their inclusion would lead to overfitted models, as there would be as many parameters to estimate as sample points.

For canopy and trunk ant species richness in the short-term experiment, we used the untransformed values of species richness at each palm. Our null models had the observed species richness of each tree as the response variable, baseline species richness as a fixed effect, plot within block within estate as nested random effects, and a Poisson error structure in our generalized linear mixed models (GLMMs). We compared these null models against equivalent models that also had treatment as a fixed effect. For the longer-term experiment, we used the same approach except that we only had plot nested within block as nested random effects because the experiment took place within one estate.

For canopy and trunk ant abundance, we added one to all abundance values and logged the resulting value (herein “logged”) and used this as our response variables. We analyzed these data using LMMs with ML estimation. Our null models for the short-term experiment contained logged baseline abundance as a fixed effect, and plot within block within estate as nested random effects. For the longer-term experiment, we used the same null models but with plot nested within block as nested random effects. In each case, we compared the null models against models also containing treatment as a fixed effect.

#### Bird and ant community composition

For each group (birds, canopy ants, trunk ants), experimental stage (before or after application of treatment) and time scale of experiment (short term or longer term), we analyzed species-abundance matrices. For birds, we square-rooted the abundance data to reduce the influence of the most abundant species on the results. To account for differences among palms in the number of occurrences of canopy and trunk ant species, we expressed the number of occurrences of each species on that palm as a proportion of total number of incidences of ant species on that palm. We then performed an ordination of our experimental plots using nonmetric multidimensional scaling (NMDS), with Bray–Curtis distances, and three dimensions (except for birds in the longer-term experiment, for which we used two dimensions) to ensure that stress was <0.1 but >0. We tested for significant differences in species composition among treatment types using analysis of similarity (ANOSIM). All community composition analyses were carried out using the vegan package (Oksanen et al. 2013).

#### Oil palm yield

We summed the total mass of fresh fruit bunches (tFFB) produced in each subplot between 6 and 15 months (inclusive) after treatment was first applied. We used this cut-off because Corley and Tinker (2003) state that there is a 5-month gap between anthesis (opening of male flowers) and production of fruit. This gap therefore ensures that anything that might affect fruit production is taken into account only in our experimental time period (we also repeated the analysis using the logged sum of tFFB for all months after treatment). We used the logged sum of tFFB as a response variable and using the lme4 package compared two LMMs (with ML estimation): a null model (with block as a random factor) and a model also containing treatment as a fixed factor.

## Results

### Epiphyte diversity

We recorded a diverse epiphyte community of 58 species – 16 species of fern (from eight families) and 42 species of angiosperm (from 23 families) (Table S1; Figs S1–S3). Sample-based species accumulation curves (Figs S1–S3) suggest that we had sampled epiphytes effectively, with all accumulation curves starting to reach their asymptote, especially for epiphytic ferns (Fig. S2). Taking the range of species richness estimates from Chao, Jack 1, Jack 2, and Bootstrap, we calculated that the total species pool of epiphytes in our sites was comprised of 69–142 species (see Table S2 for all estimates). The epiphytic fern community was estimated to contain 17–21 species, and the epiphytic angiosperm community was estimated to contain 52–126 species (Table S2).

### Species richness and abundance of birds and ants

**Figure 2:**
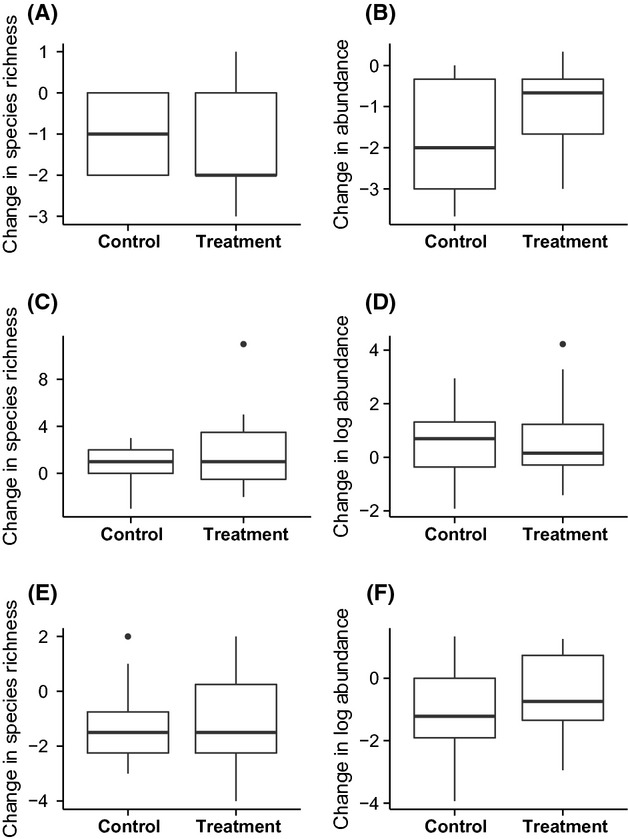
Effects of epiphyte removal in the short-term experiment on epiphyte removal treatment versus controls on (A) bird species richness and (B) bird abundance, at the plot level, and on (C) canopy ant species richness and (D) log canopy ant abundance, (E) trunk ant species richness, (F) log trunk ant species richness, at the individual palm level.

**Figure 3:**
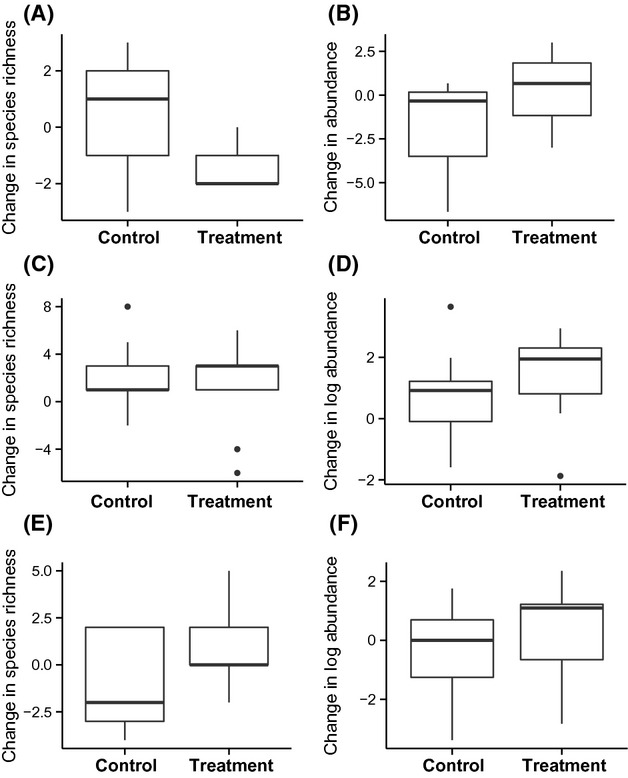
Effects of epiphyte removal in the longer-term experiment on epiphyte removal treatment versus controls on (A) bird species richness and (B) bird abundance, at the plot level, and on (C) canopy ant species richness and (D) log canopy ant abundance, (E) trunk ant species richness, (F) log trunk ant species richness, at the individual palm level.

There was little change in species richness and abundance of birds in control or treatment plots between baseline and post-epiphyte removal stages for both short-term and longer-term experiments (Figs2 and 3, respectively; Table S3; lists of sampled bird and ant species are in Tables S4 and S5, respectively). The null models in all cases had the lowest AICc value (Table1), suggesting that epiphyte removal was not an important explanatory variable.

**Table 1:** Model selection for birds and ants in the short-term and longer-term experiments. Best models are in bold

Response variable	Model	Short-term experiment		Longer-term experiment	
		AICc	ΔAICc	AICc	ΔAICc
Birds (richness)	**Null**	**14.93**	**0.00**	**21.14**	**0.00**
	Treatment	27.73	12.80	48.71	27.57
Birds (abundance)	**Null**	**5.35**	**0.00**	**17.56**	**0.00**
	Treatment	20.31	14.97	46.22	28.66
Canopy ant (richness)	**Null**	**136.37**	**0.00**	**88.19**	**0.00**
	Treatment	139.42	3.05	92.09	3.90
Canopy ant (abundance)	**Null**	**107.78**	**0.00**	**64.94**	**0.00**
	Treatment	110.62	2.84	69.57	4.63
Trunk ant (richness)	**Null**	**92.23**	**0.00**	**81.14**	**0.00**
	Treatment	95.62	3.39	84.70	3.56
Trunk ant (abundance)	**Null**	**88.16**	**0.00**	**70.44**	**0.00**
	Treatment	92.22	4.06	74.30	3.86

Median species richness and abundance of canopy ants increased over time for palms in both control and treatment plots for both short and longer time scales relative to the pre-epiphyte removal baseline (Figs2 and 3). In all cases, the null model without treatment was the better model according to AICc (Table1).

In the short-term experiment, median species richness and log abundance of trunk ants decreased for palms in both control and treatment plots after epiphyte removal (Fig.2), and again the null models were better at explaining the data (Table1). In the longer-term experiment, median log abundance increased slightly for both control and treatment palms (Fig.3). Species richness decreased for control palms and increased on removal palms, although the changes were very small (Fig.3; Table S3). However, in every case, the null models better explained these data than the models including treatment (Table1).

### Community composition of birds and ants

In the short-term experiment, there was no significant difference in community composition for birds between control and treatment plots before (*R* - −0.14, *p* - 0.90) or after (*R* - −0.012, *p* - 0.49) epiphyte removal (Figs S4–S5). The same was also true for canopy ants before (*R* - 0.023, *p* - 0.29) and after (*R* - 0.041, *p* - 0.17) epiphyte removal (Figs S6–S7) and for trunk ants before (*R* - 0.039, *p* - 0.21) and after (*R* - 0.0014, *p* - 0.48) epiphyte removal (Figs S8–S9).

In the longer-term experiment, there was no significant difference in community composition for birds between control and treatment plots before (*R* - 0.037, *p* - 0.49) or after (*R* - −0.26, *p* - 1.00) epiphyte removal (Figs S10–S11). The same was also true for canopy ants before (*R* - −0.068, *p* - 0.75) and after (*R* - −0.0089, *p* - 0.49) epiphyte removal (Figs S12–S13) and for trunk ants before (*R* - 0.038, *p* - 0.29) and after (*R* - 0.11, *p* - 0.074) epiphyte removal (Figs S14–S15). The ordination plots of Figures S8, S10, and S11 are each dominated by one outlying site, although the lack of significance means that these points do not represent statistical outliers.

### Yield

**Figure 4:**
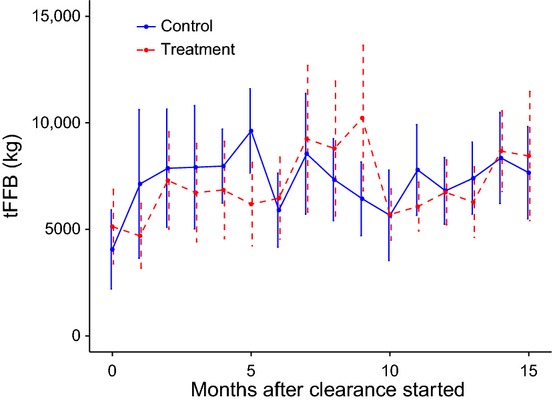
Total mass of fresh fruit bunches (tFFB), a proxy for yield, produced monthly in control and treatment (epiphyte removal) plots in our study sites within the Sabahmas estate from 0 to 15 months. Month zero is the baseline measure, and month one is the first month after the experiment began.

The models of fresh fruit production suggest that the null model, containing only block, fit the data better than the model also containing epiphyte removal treatment (Table2). The same conclusion was reached if all bunches produced after the experiment began were included (Table S6). Furthermore, the difference between control and treatment plots in each block did not show any consistent trend in fresh fruit production over time (Fig.4).

**Table 2:** Model selection for the total mass of fresh fruit bunches (tFFB) produced between 6 and 15 months (inclusive) after start of treatment (LMM). Data are from the longer-term experimental sites in Sabahmas, where epiphytes were removed over a 15-month period. Best models are in bold

Response variable	Model	AICc	ΔAICc
tFFB	**Null**	**13.65**	**0.00**
	Treatment	38.37	24.72

## Discussion

If sustainable intensification of tropical agriculture is key to reducing the loss of global biodiversity while increasing food production, then we need to clearly identify the effects of different intensification methods on both production and the taxa living within agricultural landscapes. In particular, we need to identify and discourage practices that potentially harm biodiversity without improving yield, to make agricultural intensification more sustainable. Our results show that the removal of the diverse and species rich community of epiphytes that cover oil palm trunks, a frequently promoted and applied treatment (Piggott 1980; Turner and Gillbanks 2003), has no positive effect on yield and should therefore be discouraged. While we found no negative effects of epiphyte removal on birds, trunk ants, and canopy ants, the impacts on other taxa remain unknown and could be negative.

We recorded 58 species of epiphyte, with estimators of species richness suggesting that in total there are 17–21 species of epiphytic ferns and 52–126 species of epiphytic angiosperms in the three estates we sampled. The predicted number of epiphytic ferns for our sites is lower than the number of epiphytic fern species recorded by Piggott (1980) in West Malaysia (44 species), who sampled 271 estates (at unreported sampling intensity). To our knowledge, however, ours is the first assessment of the diversity of epiphytic angiosperms in oil palm plantations.

Although mentioned as problematic in some production manuals (Turner and Gillbanks 2003), our experiments show that epiphytes have no negative impact on the mass or number of oil palm bunches, indices which both correlate well with total yield (Corley and Tinker 2003). A reason typically given to justify removal of epiphytes is that they obscure the view of the fresh fruit bunches – especially in the case of the fern *Stenochlaena palustris* – making it harder for a harvester to assess their ripeness (Corley and Tinker 2003; Turner and Gillbanks 2003), but the fact that yield was unaffected suggests that it is not a justified concern.

Our results for birds differ somewhat from a previous study (Koh 2008), which suggested that a plantation with epiphytes would have (all else being equal) 1.5 more bird species than a plantation without epiphytes. This may be because our study tested the effect of epiphytes experimentally, whereas epiphyte presence may have been correlated with other variables in Koh's study. Epiphytes are important for birds in other systems (Nadkarni and Matelson 1989), often because they provide food resources (such as hemi-epiphytic mistletoes in Australian forests (Watson and Herring 2012)) and nesting sites (Thorstrom and Roland 2000). It may be that the only surviving bird species in oil palm landscapes are generalist insectivores that do not require epiphytes as either nesting sites or food resources.

Previous studies investigating the importance of epiphytes for ants have focused on the role of epiphytes as nesting spaces for colonies, as well as microclimatic refugia within the thermally variable plantation (Fayle et al. 2010; Foster et al. 2011). However, among the epiphytes we recorded in our sites, only the bird's nest fern (*Asplenium nidus*) traps litter in such a way that it can provide large amounts of space for ants and other arthropods. Other epiphytes, which made up the vast majority of individuals in our study (Table S1), do not trap litter in this way, which may explain why their removal did not reduce the diversity and abundance of ants.

We focused our study on birds and ants. Although these are good indicator taxa (Barlow et al. 2007; Majer et al. 2007), our study does not preclude the possibility of epiphyte removal affecting other taxa. In addition to the finding that epiphytes are species rich and their removal does not benefit yield, the precautionary principle suggests that they also should not be removed in case they affect taxa we have not sampled. Furthermore, our study was limited to Sabah, Malaysian Borneo, and in other regions (e.g., Africa or the Neotropics) the relationships between epiphytes, yield, and the abundance and diversity of animals might be different. Another issue is that we only followed yield production in adult palms – we do not know if epiphytes affect yield for younger palms (in very old palms the old leaf-bases rot and fall off so there are fewer epiphytes (Piggott 1980)).

Overall our study shows that reduction of habitat complexity via epiphyte removal within oil palm does not necessarily improve yield, and we must urgently identify features of habitat complexity in other systems that can be maintained without loss of yield. Unless we explicitly test the benefits to yield and costs to biodiversity of different intensive management practices, we risk a homogenization of habitat complexity – and potentially biodiversity – for no added yield benefits.

## Supporting Information

**Table S1.** List of epiphytes recorded in our study. **Table S2.** Different estimators of total species richness for our sites. **Table S3.** Mean species richness and abundance of birds, canopy ants and trunk ants in control and treatment plots, before and after treatment was applied, for short-term and longer-term experiments. **Table S4.** List of bird species recorded. **Table S5**. List of ant morphospecies recorded. **Table S6.** Model selection for yield (if all data after clearance began is included). **Figure S1.** Species accumulation curve for all epiphytes. **Figure S2.** Species accumulation for epiphytic ferns. **Figure S3.** Species accumulation curves for epiphytic angiosperms. **Figure S4.** Ordination of bird communities before treatment (short-term experiment). **Figure S5.** Ordination of bird communities after treatment (short-term experiment). **Figure S6.** Ordination of canopy ant communities before treatment (short-term experiment). **Figure S7.** Ordination of canopy ant communities after treatment (short-term experiment). **Figure S8**. Ordination of trunk communities before treatment (short-term experiment). **Figure S9**. Ordination of trunk ant communities after treatment (short-term experiment). **Figure S10**. Ordination of bird communities before treatment (longer-term experiment). **Figure S11**. Ordination of bird communities after treatment (longer-term experiment). **Figure S12.** Ordination of canopy ant communities before treatment (longer-term experiment). **Figure S13**. Ordination of canopy ant communities after treatment (longer-term experiment). **Figure S14**. Ordination of trunk communities before treatment (longer-term experiment). **Figure S15**. Ordination of trunk ant communities after treatment (longer -term experiment).
