# Supplementary material for: Retaining biodiversity in intensive farmland: epiphyte removal in oil palm plantations does not affect yield
Source: Ecol Evol. 2015 Apr 29;5(10):1944–54. doi: 10.1002/ece3.1462 (PMC4449750; doi:10.1002/ece3.1462)
Supplement: Supplementary file 1 [file ece30005-1944-sd1.docx]

**Supporting Information**

**Retaining biodiversity in intensive farmland: epiphyte removal in oil palm plantations does not affect yield**

Graham W Prescott^1^*, David P Edwards^2,3^, William A Foster^1^

^1^Department of Zoology, University of Cambridge, Cambridge, Cambridgeshire, United Kingdom

^2^Department of Animal and Plant Sciences, University of Sheffield, Sheffield, South Yorkshire, United Kingdom

^3^Centre for Tropical Environmental and Sustainability Science (TESS) and School of Marine and Tropical Biology, James Cook University, Cairns, Queensland, Australia

* Corresponding author. Email: [grahamprescott@gmail.com](mailto:grahamprescott@gmail.com)

Contents:

Table S1 – list of epiphytes recorded in our study

Table S2 – different estimators of total species richness for our sites

Table S3 – mean species richness and abundance of birds, canopy ants and trunk ants in control and treatment plots, before and after treatment was applied, for short-term and longer-term experiments.

Table S4 – List of bird species recorded

Table S5 – List of ant morphospecies recorded

Table S6 – model selection for yield (if all data after clearance began is included)

Figure S1 – species accumulation curve for all epiphytes

Figure S2 – species accumulation for epiphytic ferns

Figure S3 – species accumulation curves for epiphytic angiosperms

Figure S4 – ordination of bird communities before treatment (short-term experiment)

Figure S5 – ordination of bird communities after treatment (short-term experiment)

Figure S6 – ordination of canopy ant communities before treatment (short-term experiment)

Figure S7 – ordination of canopy ant communities after treatment (short-term experiment)

Figure S8- ordination of trunk communities before treatment (short-term experiment)

Figure S9 – ordination of trunk ant communities after treatment (short-term experiment)

Figure S10 – ordination of bird communities before treatment (longer-term experiment)

Figure S11 – ordination of bird communities after treatment (longer-term experiment)

Figure S12 – ordination of canopy ant communities before treatment (longer-term experiment)

Figure S13 – ordination of canopy ant communities after treatment (longer-term experiment)

Figure S14- ordination of trunk communities before treatment (longer-term experiment)

Figure S15 – ordination of trunk ant communities after treatment (longer -term experiment)

Table S1 – List of epiphytes recorded in our study, with asterisk (*) denoting species known to be non-native. All names follow those accepted in *The Plant List* (<http://www.theplantlist.org/>); names listed as unresolved are marked §

| Division | Family name | Species |
| --- | --- | --- |
| Angiosperm | Acanthaceae | *Asystasia gangetica §* |
|  |  | *Justicia gendarussa* |
|  | Amaranthacaea | *Alternanthera sessilis* |
|  | Araceae | *Alocasia longiloba* |
|  |  | *Alocasia princeps* |
|  |  | *Alocasia sarawakensis* |
|  |  | *Homalomena sp. 1* |
|  | Arecaceae | *Elaeis guineensis ** |
|  | Asteraceae | *Ageratum conyzoides ** |
|  |  | *Chromolaena odorata ** |
|  |  | *Cyanthillium cinereum* |
|  |  | *Elephantopus scaber* |
|  |  | *Mikania micrantha ** |
|  | Costaceae | *Cheilocostus speciosus* |
|  | Cyperaceae | *Rhynchospora colorata ** |
|  |  | *Scleria ciliaris* |
|  | Dioscoreaceae | *Dioscorea oppositifolia ** |
|  | Euphorbaceae | *Macaranga tanarius* |
|  |  | *Mallotus korthalsii* |
|  | Fabacaea | *Mucuna bracteata ** |
|  |  | *Spatholobus sp. 1* |
|  | Gentianaceae | *Fagraea cuspidata* |
|  | Gesneriaceae | *Aeschynanthus sp. 1* |
|  | Lamiaceae | *Callicarpa longifolia* |
|  | Linderniaceae | *Torenia sp.* |
|  | Melastomataceae | *Clidemia hirta ** |
|  | Moraceae | *Ficus heterophylla* |
|  |  | *Ficus obscura* |
|  |  | *Ficus sp. 1* |
|  |  | *Ficus sp. 2* |
|  | Orchidaceae | *Liparis latifolia* |
|  | Phyllanthaceae | *Glochidion rubrum* |
|  |  | *Phyllanthus niruri* |
|  | Piperaceae | *Peperomia pellucida* |
|  | Rubiaceae | *Nauclea subdita* |
|  |  | *Uncaria sp. 1* |
|  | Solanaceae | *Solanum frutescens *§* |
|  | Urticaceae | *Dendrocnide sp. 1* |
|  |  | *Leucosyke capitellata §* |
|  |  | *Pipturus argenteus* |
|  |  | *Poikilospermum suaveolens* |
|  | Vitaceae | *Leea indica* |
| Pteridophyte | Aspleniaceae | *Asplenium longissimum * §* |
|  |  | *Asplenium macrophyllum* |
|  |  | *Asplenium nidus* |
|  |  | *Asplenium nitidum §* |
|  | Blechnaceae | *Stenochlaena palustris* |
|  | Davalliaceae | *Davallia denticulata* |
|  | Nephrolepidaceae | *Nephrolepis biserrata* |
|  | Polypodiaceae | *Goniophlebium percussum* |
|  |  | *Goniophlebium sp. 1* |
|  |  | *Neocheiropteris sarawakense* |
|  |  | *Phymatosorus scolopendria* |
|  | Tectariaceae | *Heterogonium pinnatum* |
|  | Thelypteridaceae | *Sphaerostephanos porphyricola §* |
|  | Vittariaceae | *Vittaria elongata* |
|  |  | *Vittaria ensiformis* |
|  | NA | Unidentified fern, sp. 1 |

Table S2 different estimators of total species richness for our sites (plus or minus standard error). Note that no standard error is available for Jack 2.

| Division/Measure | Number of Species | Number of samples | Chao | Jack 1 | Jack 2 | Bootstrap |
| --- | --- | --- | --- | --- | --- | --- |
| All epiphytes | 58 | 80 | 142.1 ± 49.7 | 86.6 ± 6.3 | 110.1 | 69.4 ± 3.2 |
| Epiphytic ferns | 16 | 80 | 20.5 ± 7.2 | 18.9 ± 1.7 | 20.9 | 17.3 ± 1.0 |
| Epiphytic angiosperms | 42 | 80 | 126.5 ± 54.5 | 67.7 ± 5.9 | 110.1 | 52.1 ± 3.0 |

Table S3 – mean and standard error for each of the variables we measured in the short-term and longer-term experiments, in control and treatment plots, and before and after application of treatment. For richness the response is the number of species observed, and for abundance it is the total number of individuals observed.

| Response | Stage | Short-term experiment | | Longer-term experiment | |
| --- | --- | --- | --- | --- | --- |
|  |  | Control | Treatment | Control | Treatment |
| Birds (richness) | Before | 10.00 ± 0.45 | 9.40 ± 0.68 | 9.00 ± 1.15 | 8.67 ± 0.88 |
|  | After | 9.00 ± 0.55 | 8.20 ± 0.58 | 9.33 ± 0.67 | 7.33 ± 1.20 |
| Birds (abundance) | Before | 12.07 ± 0.64 | 11.40 ± 0.24 | 12.56 ± 1.57 | 11.33 ± 0.96 |
|  | After | 10.27 ± 0.16 | 10.33 ± 0.41 | 10.44 ± 0.73 | 11.56 ± 1.39 |
| Canopy ant (richness) | Before | 2.73 ± 0.45 | 3.27 ± 0.37 | 2.22 ± 0.60 | 3.11 ± 1.20 |
|  | After | 3.67 ± 0.36 | 5.07 ± 0.64 | 4.22 ± 0.86 | 4.44 ± 0.63 |
| Canopy ant (abundance) | Before | 30.07 ± 11.14 | 22.53 ± 7.56 | 88.78 ± 72.84 | 18.44 ± 9.25 |
|  | After | 50.67 ± 18.61 | 46.27 ± 11.23 | 47.11 ± 15.04 | 47.89 ± 17.88 |
| Trunk ant (richness) | Before | 3.58 ± 0.40 | 3.08 ± 0.54 | 4.11 ± 0.70 | 3.00 ± 0.41 |
|  | After | 2.33 ± 0.45 | 1.92 ± 0.42 | 3.33 ± 0.58 | 3.89 ± 0.61 |
| Trunk ant (abundance) | Before | 50.00 ± 15.98 | 21.33 ± 8.56 | 16.22 ± 5.71 | 14.78 ± 6.86 |
|  | After | 14.33 ± 5.57 | 10.25 ± 3.48 | 18.00 ± 9.22 | 26.11 ± 9.98 |

Table S4 – List of bird species recorded in study plots.

| **Latin name** | **English name** | **Family** |
| --- | --- | --- |
| *Amauromis phoenicurus* | white-breasted waterhen | Rallidae |
| *Cacomantis merulinus* | plaintive cuckoo | Cuculidae |
| *Centropus sinensis* | greater coucal | Cuculidae |
| *Chalcophaps indica* | emerald dove | Columbidae |
| *Copsychus saularis* | oriental magpie-robin | Muscicapidae |
| *Copsychus stricklandii* | white-crowned shama | Muscicapidae |
| *Corvus enca* | slender-billed crow | Corvidae |
| *Gracula religiosa* | hill mynah | Sturnidae |
| *Macronous bornensis* | bold-striped tit-babbler | Timaliidae |
| *Orthotomus ruficeps* | ashy tailorbird | Cisticolidae |
| *Orthotomus sericeus* | rufous-tailed tailorbird | Cisticolidae |
| *Passer montanus* | eurasian tree sparrow | Passeridae |
| *Prinia flaviventris* | yellow-bellied prinia | Cisticolidae |
| *Pycnonotus goiavier* | yellow-vented bulbul | Pycnonotidae |
| *Rhipidura javanica* | pied fantail | Rhipiduridae |
| *Spilopelia chinensis* | spotted dove | Columbidae |
| *Spilornis cheela* | crested serpent eagle | Accipitridae |
| *Todiramphus chloris* | collared kingfisher | Alcedinidae |

Table S5 – List of ant morpho-species recorded in study plots

| *Anoplolepis gracilipes* | *Leptogenys sp. 2* | *Pheidologeton sp. 1* |
| --- | --- | --- |
| *Camponotus sp. 1* | *Lophomyrmex bedoti* | *Plagiolepis alluaudi* |
| *Camponotus sp. 2* | *Monomorium sp. 2* | *Plagiolepis sp. 2* |
| *Camponotus sp. 3* | *Monomorium sp. 3* | *Platythyrea sp. 1* |
| *Camponotus sp. 4* | *Monomorium sp. 4* | *Platythyrea sp. 2* |
| *Cardiocondyla sp. 1* | *Monomorium sp. 5* | *Platythyrea sp. 3* |
| *Cardiocondyla sp. 2* | *Monomorium sp. 6* | *Polyrachis sp. 1* |
| *Carebara sp. 3* | *Monomorium sp. 7* | *Polyrachis sp. 2* |
| *Carebara sp. 5* | *Myrmicaria sp. 1* | *Polyrachis sp. 3* |
| *Centromyrmex sp. 1* | *Nylanderia sp. 2* | *Polyrachis sp. 4* |
| *Cerapachys sp. 1* | *Nylanderia sp. 3* | *Ponera sp. 1* |
| *Cerapachys sp. 2* | *Odontomachus sp. 1* | *Ponera sp. 2* |
| *Crematogaster sp. 1* | *Oecophylla smaragdina* | *Prenolepis sp. 3* |
| *Crematogaster sp. 2* | *Pachycondyla sp. 1* | *Prenolepis sp. 4* |
| *Crematogaster sp. 3* | *Paraparatrechina sp. 1* | *Rhoptromyrmex sp. 1* |
| *Crematogaster sp. 4* | *Paraparatrechina sp. 2* | *Solenopsis sp. 1* |
| *Crematogaster sp. 5* | *Paratrechina longicornis* | *Strumigenys sp. 2* |
| *Crematogaster sp. 7* | *Pheidole sp. 1* | *Tapinoma melanocephalum* |
| *Crematogaster sp. 8* | *Pheidole sp. 11* | *Tapinoma sp. 2* |
| *Diacamma sp. 1* | *Pheidole sp. 2* | *Technomyrmex sp. 1* |
| *Dolichoderus sp. 1* | *Pheidole sp. 3* | *Technomyrmex sp. 2* |
| *Forelophilus sp. 2* | *Pheidole sp. 4* | *Tetramorium sp. 1* |
| *Forelophilus sp. 1* | *Pheidole sp. 5* | *Tetramorium sp. 2* |
| *Forelophilus sp. 2* | *Pheidole sp. 6* | *Tetramorium sp. 3* |
| *Gnamptogenys sp. 1* | *Pheidole sp. 7* | *Tetramorium sp. 4* |
| *Hypoponera sp. 1* | *Pheidole sp. 8* | *Tetramorium sp. 5* |
| *Leptogenys sp. 1* | *Pheidole sp. 9* | *Vollenhovia sp. 1* |

Table S6 – AICc for LMMs with the logged sum of tFFB (total mass of fresh fruit bunches produced) for all post-clearance months.

| Model | AICc | delta AICc |
| --- | --- | --- |
| **Null** | **10.23** | **0.00** |
| Treatment | 34.01 | 23.78 |


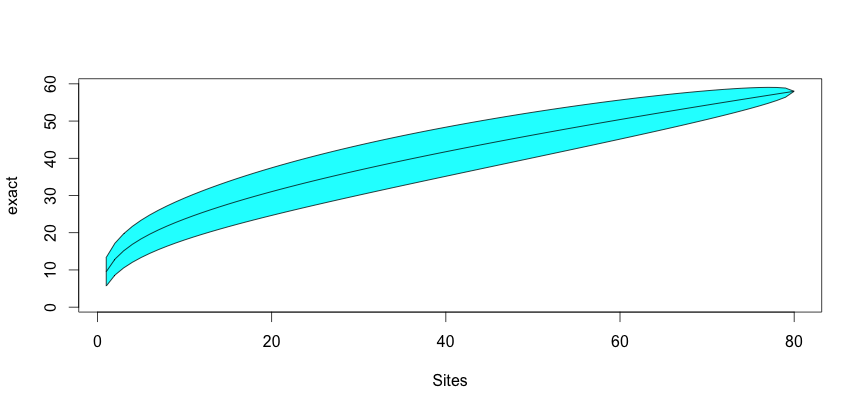


Figure S1 – species accumulation curve for all epiphytes (with 95% confidence interval).
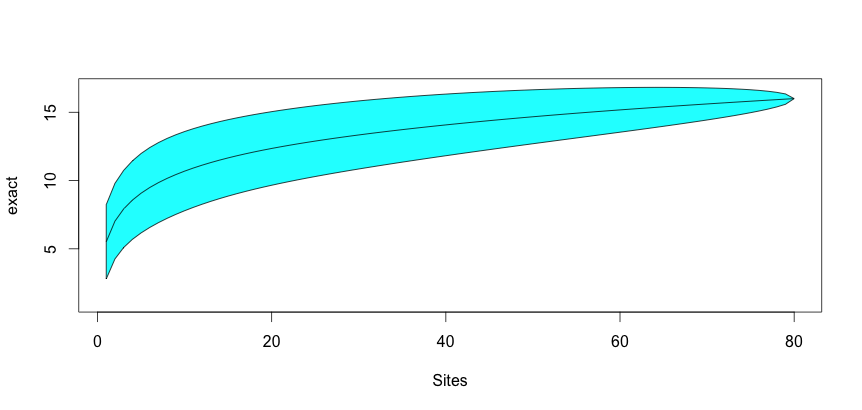


Figure S2 – species accumulation curve for epiphytic ferns (with 95% confidence interval).
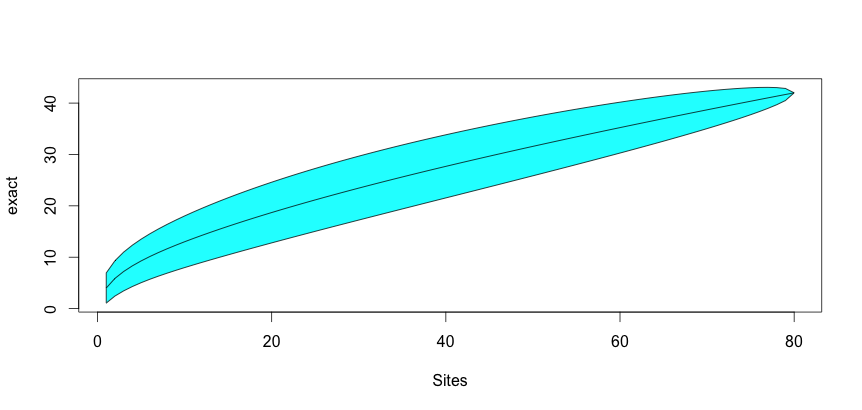


Figure S3 – species accumulation curve for epiphytic angiosperms (with 95% confidence interval).


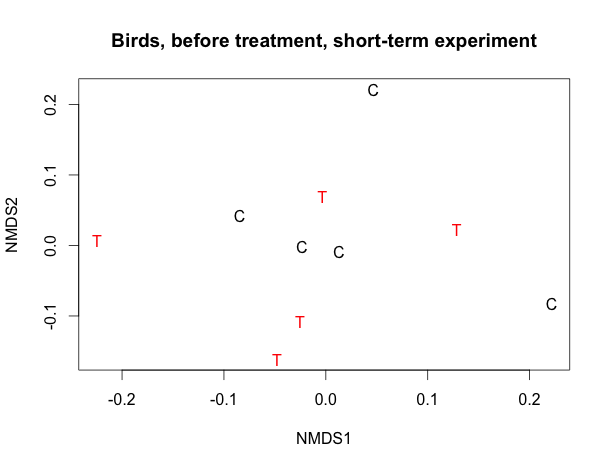


Supplementary figure 4 – ordination of sites using NMDS (with Bray-Curtis distance) in the short-term experiment, according to bird community composition before the treatment was applied. C= control plots, T=treatment (epiphyte removal) plots.


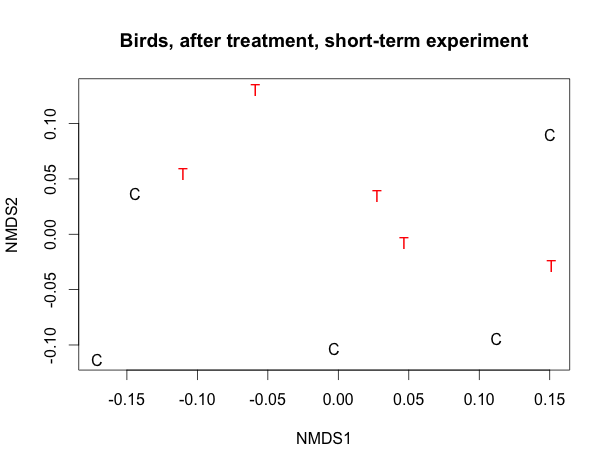


Supplementary figure 5 – ordination of sites using NMDS (with Bray-Curtis distance) according to bird community composition 2 months after treatment was applied. C= control plots, T=treatment (epiphyte removal) plots.


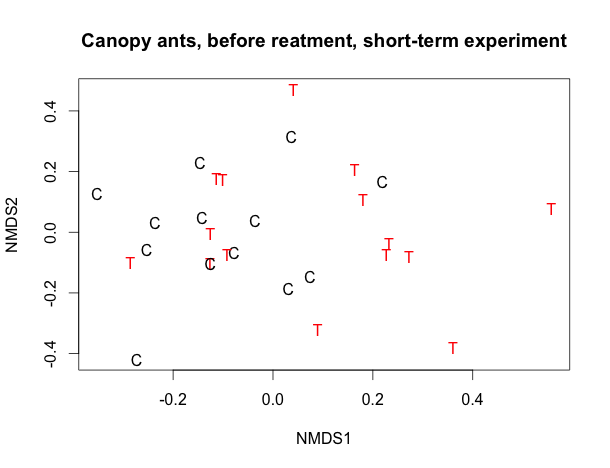


Supplementary figure 6 – ordination of sites using NMDS (with Bray-Curtis distance) in the short-term experiment according to canopy ant community composition before the treatment was applied. C= control palms, T=treatment (epiphyte removal) palms.


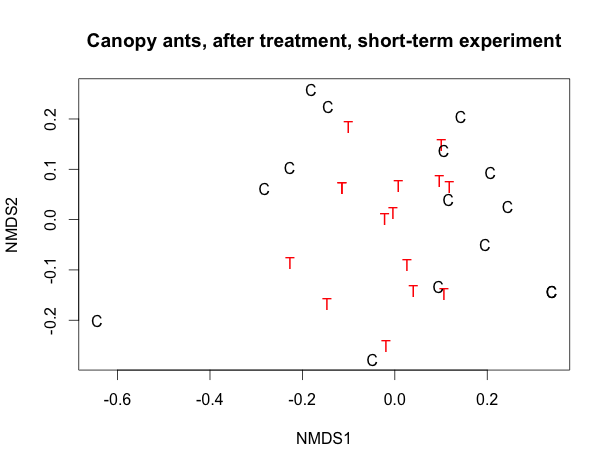


Supplementary figure 7 – ordination of sites using NMDS (with Bray-Curtis distance) in the short term experiment according to canopy ant community composition 2 months after treatment was applied. C= control palms, T=treatment (epiphyte removal) palms.


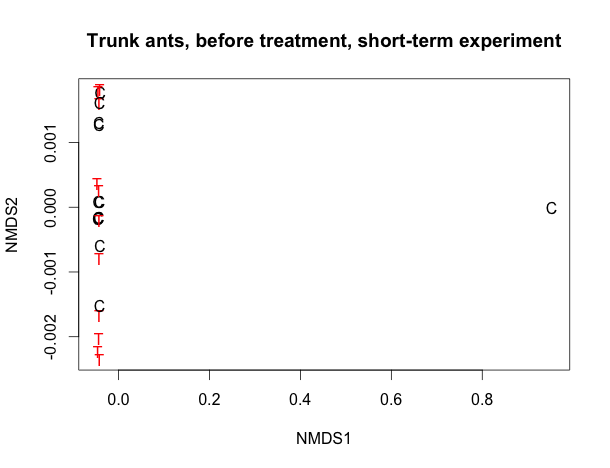


Supplementary figure 8 – ordination of sites using NMDS (with Bray-Curtis distance) in the short-term experiment according to trunk ant community composition before the treatment was applied. C= control palms, T=treatment (epiphyte removal) palms.


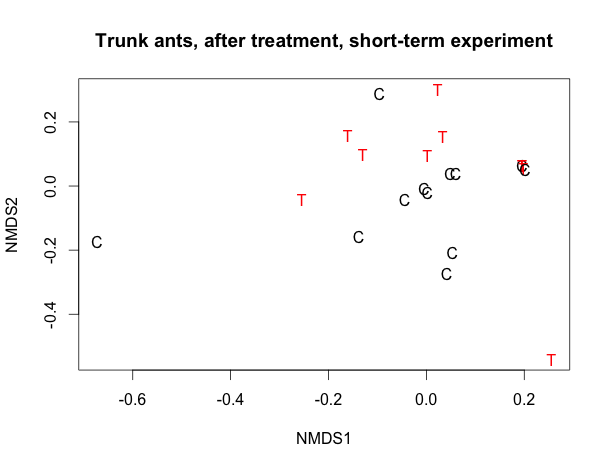


Supplementary figure 9 – ordination of sites using NMDS (with Bray-Curtis distance) in the short-term experiment according to trunk ant community composition 2 months after treatment was applied. C= control palms, T=treatment (epiphyte removal) palms.


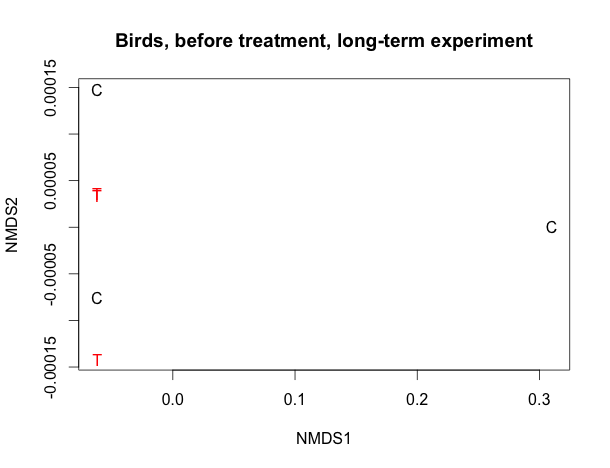


Supplementary figure 10 – ordination of sites using NMDS (with Bray-Curtis distance) in the longer-term experiment, according to bird community composition before the treatment was applied. C= control plots, T=treatment (epiphyte removal) plots.


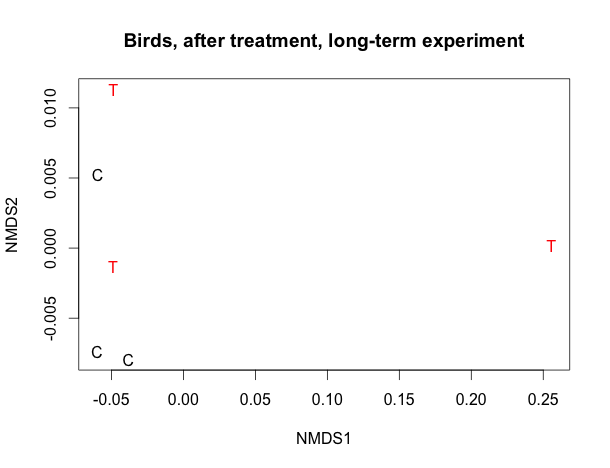


Supplementary figure 11 – ordination of sites using NMDS (with Bray-Curtis distance) in the longer-term experiment according to bird community composition 2 months after treatment was applied. C= control plots, T=treatment (epiphyte removal) plots.


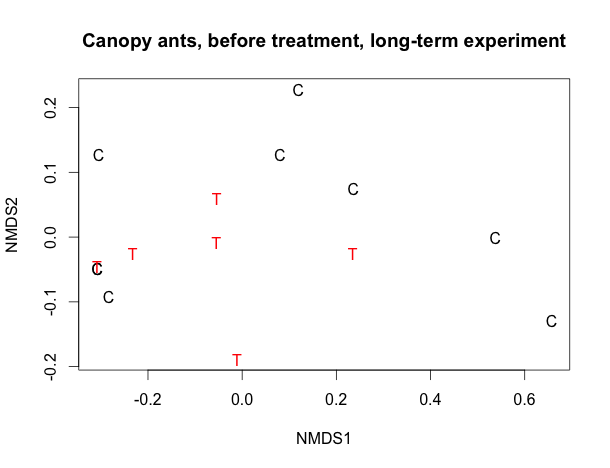


Supplementary figure 12 – ordination of sites using NMDS (with Bray-Curtis distance) in the longer-term experiment according to canopy ant community composition before the treatment was applied. C= control palms, T=treatment (epiphyte removal) palms.


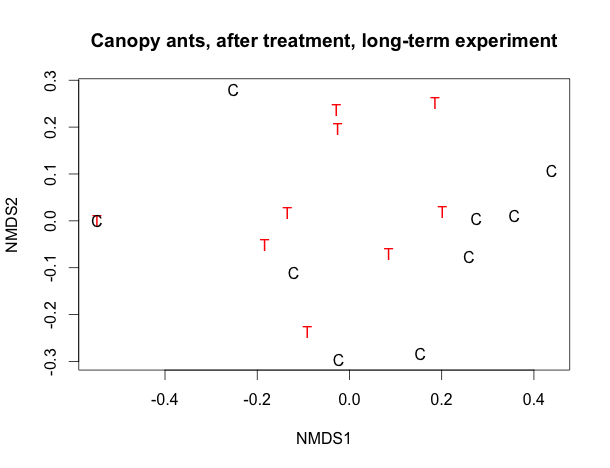


Supplementary figure 13 – ordination of sites using NMDS (with Bray-Curtis distance) in the longer-term experiment according to canopy ant community composition 2 months after treatment was applied. C= control palms, T=treatment (epiphyte removal) palms.


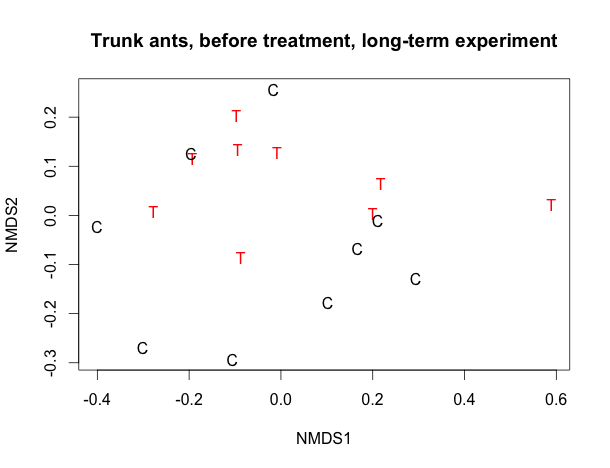


Supplementary figure 14 – ordination of sites using NMDS (with Bray-Curtis distance) in the longer-term experiment according to trunk ant community composition before the treatment was applied. C= control palms, T=treatment (epiphyte removal) palms.


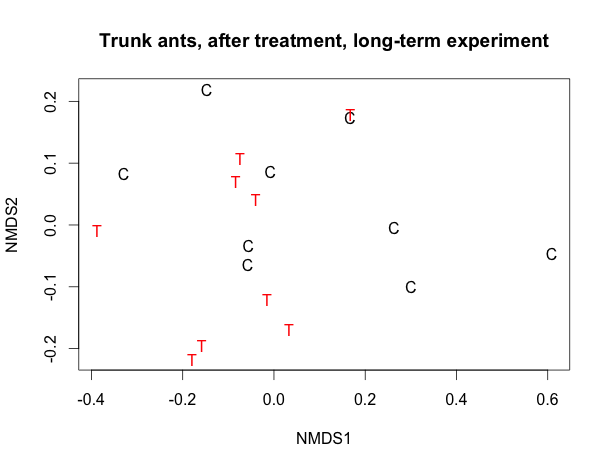


Supplementary figure 15 – ordination of sites using NMDS (with Bray-Curtis distance) in the longer-term experiment according to trunk ant community composition 2 months after treatment was applied. C= control palms, T=treatment (epiphyte removal) palms.
